# Molecular Disorder in Crystalline Thin Films of an Asymmetric BTBT Derivative

**DOI:** 10.1021/acs.chemmater.0c04725

**Published:** 2021-02-09

**Authors:** Sebastian Hofer, Johanna Unterkofler, Martin Kaltenegger, Guillaume Schweicher, Christian Ruzié, Adrián Tamayo, Tommaso Salzillo, Marta Mas-Torrent, Alessandro Sanzone, Luca Beverina, Yves Henry Geerts, Roland Resel

**Affiliations:** † Institute of Solid State Physics, Graz University of Technology, Petersgasse 16, Graz 8010, Austria; ‡ Laboratoire de Chimie des Polymères, Faculté des Sciences, Université Libre de Bruxelles, Campus Plaine, CP206/01 - Boulevard du, Triomphe, Bruxelles 1050, Belgium; § Institut de Ciència de Materials de Barcelona, ICMAB-CSIC, Campus de la UAB, 08193 Bellaterra, Spain; ∥ Department of Materials Science, University of Milano-Bicocca, Via Roberto Cozzi, 55, Milano 20125, Italy; ⊥ Laboratoire de Chimie des Polymères, Faculté des Sciences, International Solvay Institutes of Physics and Chemistry, Université Libre de Bruxelles, Campus Plaine, CP206/01 - Boulevard du Triomphe, Brussels 1050, Belgium

## Abstract

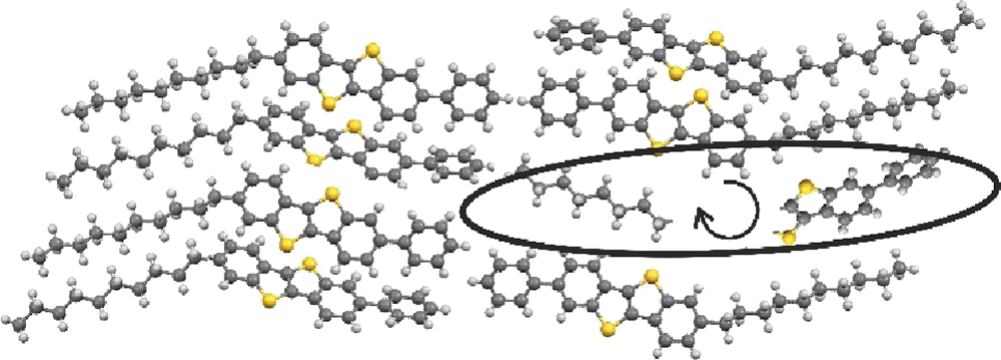

The molecule 2-decyl-7-phenyl-[1]benzothieno[3,2-b][1]benzothiophene (Ph-BTBT-10) is an organic semiconductor with outstanding performance in thin-film transistors. The asymmetric shape of the molecule causes an unusual phase behavior, which is a result of a distinct difference in the molecular arrangement between the head-to-head stacking of the molecules versus head-to-tail stacking. Thin films are prepared at elevated temperatures by crystallization from melt under controlled cooling rates, thermal-gradient crystallization, and bar coating at elevated temperatures. The films are investigated using X-ray diffraction techniques. Unusual peak-broadening effects are found, which cannot be explained using standard models. The modeling of the diffraction patterns with a statistic variation of the molecules reveal that a specific type of molecular disorder is responsible for the observed peak-broadening phenomena: the known head-to-head stacking within the crystalline phase is disturbed by the statistic integration of reversed (or flipped) molecules. It is found that 7–15% of the molecules are integrated in a reversed way, and these fractions are correlated with cooling rates during the sample preparation procedure. Temperature-dependent in situ experiments reveal that the defects can be healed by approaching the transition from the crystalline state to the *smectic E* state at a temperature of 145 °C. This work identifies and quantifies a specific crystalline defect type within thin films of an asymmetric rodlike conjugated molecule, which is caused by the crystallization kinetics.

## Introduction

The crystal structure is of fundamental importance to understand the properties of organic semiconductors. The long-range order of the molecules is a defined way to describe molecular packing so that electronic and phonon band structures can be calculated. The distribution of electrons in terms of their energies and their apparent momenta is the basis to understand charge transport in these materials.^1^ However, the deviation from the ideal crystal lattice is also important to understand application-relevant properties of organic semiconductors.^2^ There exists a variety of structural defects that cause variations from the ideality.^3^ Chemical defects arise from substitutional molecules^4^ or physical defects because of the distortion of periodic lattices like vacancies, dislocation lines, or stacking faults.^5,6^ Optical properties as well as charge transport within organic semiconductors are highly affected by defects.^7−9^ Structural defects at a considerably larger length scales are the crystal size and crystal mosaicity associated with grain boundaries between single-crystalline domains.^10^

The solution of crystal structures is a widely used and highly advanced technique mainly based on X-ray diffraction (XRD).^11^ The characterization of structural defects is considerably more difficult, and direct observations using microscopy methods are the most successful techniques.^12^ However, microscopy methods are often difficult to perform and give only selective results; therefore, the use of integral methods is preferred. Therefore, X-ray scattering techniques are preferred, but the nature of the structural defects is often difficult to identify.^13^

There are a number of effects in the XRD pattern, which can be assigned to defects. Diffuse scattering (the scattering apart from Bragg peaks) appears because defects break the long-range periodicity of a crystal structure.^14,15^ The peak broadening of Bragg peaks is one quite well-developed tool to characterize the deviation from the ideal infinitely extended perfect crystal. The two contributions for peak broadening are microstrains (root-mean-square of the variations in the lattice parameters) and the crystal size (size of the ideal crystal in the direction of the scattering vector). The separation of these two effects is possible with Williamson–Hall plots.^16^ This method has successfully been applied to thin films of organic semiconductors.^17,18^

**Figure 1:**
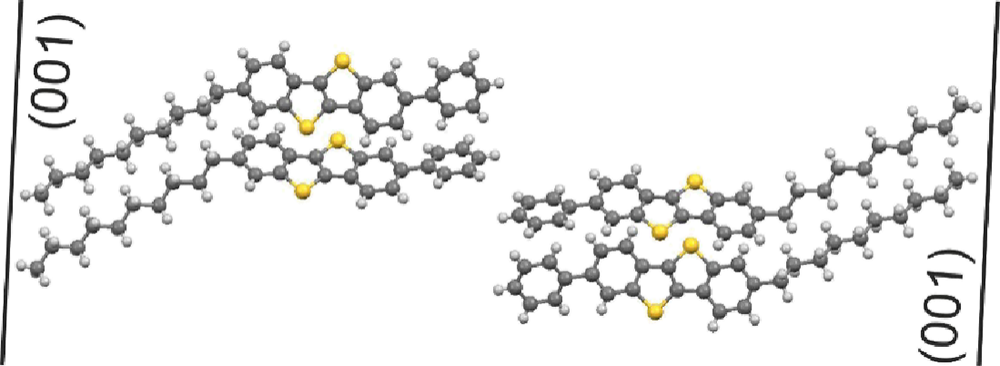
Packing of the molecule Ph-BTBT-10 within the crystalline state. Bilayers are formed with head-to-head arrangement of the molecules. Additionally, the crystallographic (001) planes are drawn.

The molecule 2-decyl-7-phenyl-[1]benzothieno[3,2-b][1]benzothiophene (Ph-BTBT-10) is a recently developed organic semiconductor that exhibits excellent performance in thin-film transistors.^19,20^ The asymmetric nature of the molecule, there is a phenyl ring at one terminal end of the aromatic benzothieno–benzothiophene core and a decyl chain at the other terminal end, causes a specific phase behavior as a function of temperature. Starting from the elevated temperatures, a phase transition from the isotropic liquid to the *smectic A* phase appears at 223 °C and subsequently a transition to the *smectic E* phase at 210 °C.^19,21^ The transition between the liquid crystalline state and the crystalline state is accompanied with strong retardation effects: the onset of crystallization appears at around 100 °C, while the melting of the crystalline state occurs at a temperature of about 143 °C. Within the crystalline state, the molecules form a double-layer herringbone structure: the layers are formed from separated alkyl chains and the aromatic parts of the molecule (compare Figure 1).^22^ The molecules of neighboring layers are arranged in a head-to-head arrangement so that the BTBT core together with the terminal phenyl ring points toward each other.

Thin film preparation for transistor applications is performed at elevated temperatures because the moderately ordered *smectic E* phase is expected as a prestage of crystallization.^23,24^ The present work shows that a molecular disorder is obtained when the thin films of the molecule Ph-BTBT-10 are processed from elevated temperatures. The obtained crystalline packing is substantially disordered, and the type of defect could be identified. The defects could be healed by heat treatment at temperatures close to phase transition to the liquid crystalline state.

## Experimental Section

One batch of the molecule Ph-BTBT-10 was synthetized according to the strategy recently published.^19^ The first sample series was deposited on 20 × 20 mm^2^ Menzel glass substrates by drop-casting from an 8 g/L solution. Toluene was used as the solvent. The thin film samples were heated up to 245 °C, a temperature well above the melting point of the material.^19^ In a subsequent step, the samples were cooled to room temperature; defined cooling rates between 0.8 °C/min up to 30 °C/min were chosen. The heat treatment was performed with the commercially available domed hot stage DHS900, Anton Paar.^25^

The second sample series was also based on drop-casted thin films from toluene solutions, but the heat treatment was performed by thermal-gradient crystallization.^26^ The setup consists of two independent heating stages (hot end and cold end) separated by a gap of 2.5 mm. A mechanical arm allows the displacement of the sample from the hot end to the cold end at a constant translation velocity, chosen in between 1 and 20 μm/s. For this purpose, the thin film was covered using an additional glass slide. The stack (substrate/ thin film/top glass) was heated to 245 °C for the complete melting of the thin film. Crystallization takes place by a translational movement toward the low-temperature zone which was maintained at 75 °C, a temperature well below the crystallization temperature of Ph-BTBT-10. The used system is a Linkam GS350 temperature gradient heating stage combined with a Nikon Eclipse 80i polarized light microscope.

The third sample series was prepared by shear crystallization^27^ on oxidized silicon wafers by bar-assisted meniscus shearing, following the previously reported methodology.^28^ Solutions of Ph-BTBT-10 and blends of Ph-BTBT-10 with polystyrene (PS) in a ratio Ph-BTBT-10:PS 2:1 in chlorobenzene and o-xylene with a concentration of 2% and 2.5% w/w, respectively, were prepared. The addition of a binding insulating polymer has been reported to promote the crystallization of the organic semiconductor and improve thin-film homogeneity.^29^ Deposition was performed at elevated substrate temperatures of 105 and 110 °C for chlorobenzene and o-xylene, respectively.

Specular XRD was performed with an PANalytical Empyrean system using a sealed copper tube together with a multilayer mirror for generating a parallelized and monochromatized primary X-ray beam. A wavelength of 1.542 Å was used. The scattered intensity was detected with a PIXcel detector operating in a one-dimensional (1D) mode for long-range measurements and as a point detector for short-range measurements. The diffraction pattern was converted into a reciprocal space using the equation , with *q* as the length of the scattering vector, λ as the used wavelength, and 2θ as the angle between the primary and the scattered X-ray beam. The peak parameters were evaluated by subtracting the experimental background and fitting a Gaussian curve to determine peak positions and peak widths; the peak widths are given as full width at half maximum. In situ temperature-dependent measurements were performed using the high-temperature attachment DHS900,^25^ setting the temperature and waiting for 10 min to let the system equilibrate before taking the measurements. Subsequently, the temperature is increased in steps of 2 °C close to the phase transitions and with a step size of 10 °C elsewhere.

The calculations of specular diffraction patterns were performed by the Fourier transforms of electron densities. The electron density was modeled by the number of electrons for each atom in a single molecule of Ph-BTBT-10 along the 001 direction, with a width based on the atomic form factor for each element. Based on the known crystal structure of Ph-BTBT-10, a layered structure was assumed with a defined layer distance of 26.5 Å. The thickness of the layer results from the length of the molecules together with their molecular packing of a herringbone type. The electron density distribution across the layers was chosen as nonperiodic by molecular disorders. To include the disorder, each layer is stacked on top of the previous layer with a certain probability of being either in a head-to-head or head-to-tail fashion. The fraction of molecular disorder *p* is defined by the fraction of molecules with a head-to-head orientation. A molecular disorder parameter *p = 0* appears, if the number of up-right standing molecules is equal to the downward oriented molecules. A number of repeating units is set at 30 to be in best agreement with the experimentally prepared samples. This modeling is repeated up to 200 times, and the resulting Fourier signal is averaged to generate a smooth calculated diffraction signal.

Grazing incidence X-ray diffraction (GIXRD) was performed at the XRD1 beamline, Elettra, Trieste. The primary X-ray beam with a wavelength of 1.400 Å and a size of 500 × 500 μm^2^ enclosed an incident angle of 1.5° with the sample surface. The diffracted beams were detected with a stationary Pilatus 2 M detector. The diffraction pattern was converted into the reciprocal space using the software *GIDVis*;^30^ the calculation of peak positions and peak intensities from the known crystal structure of Ph-BTBT-10 was performed by the module *crystal* available within the *GIDVis* package.

## Results

**Figure 2:**
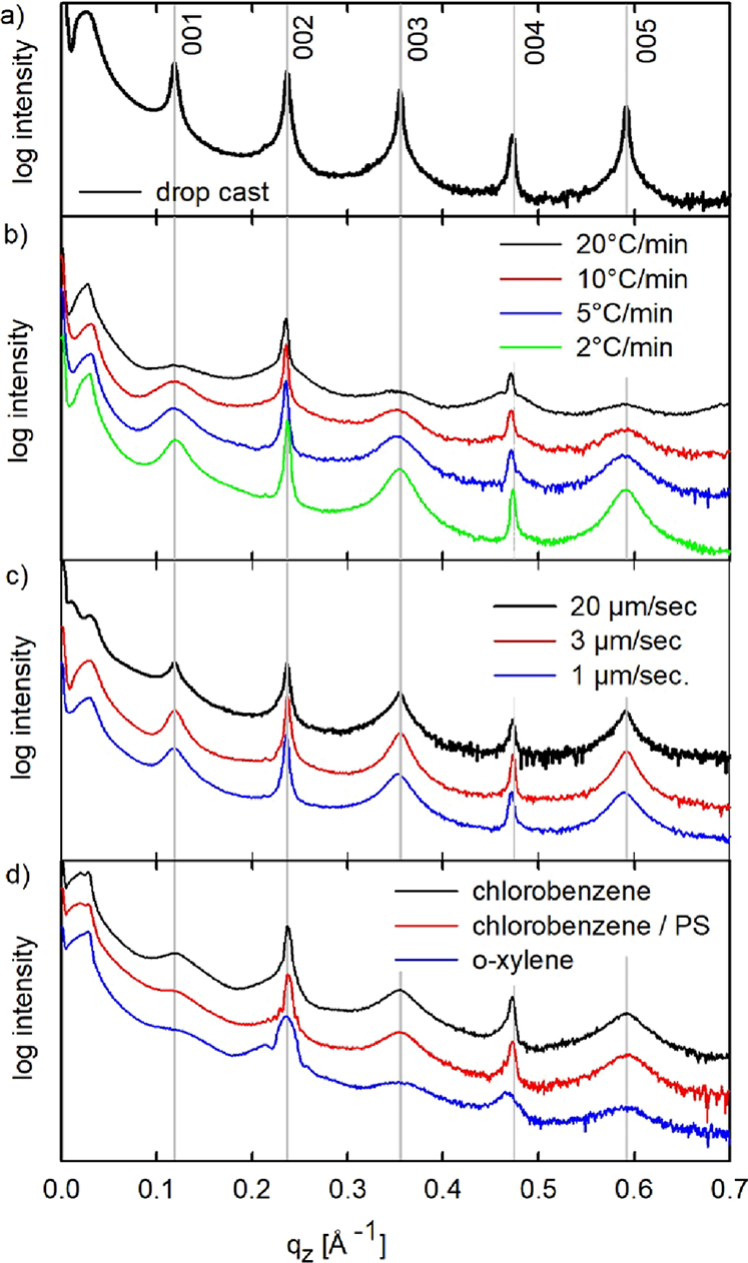
Specular XRD of thin films of the molecule Ph-BTBT-10. (a) an untreated film, (b) films obtained from the melt by defined cooling rates, (c) thermal-gradient crystallization from different withdrawal velocities, and (d) shear crystallization using different solvents with and without using polystyrene (PS) as the binding polymer.

All the three sample series were investigated by specular XRD. The first sample series is based on the drop-casted thin films. The result of an untreated drop-casted film is shown in Figure 2a. The diffraction pattern reveals defined diffraction peaks which could be indexed on the basis of the known crystal structure of Ph-BTBT-10.^22^ The peak located at 0.118 Å^–1^ can be assigned to the 001 Bragg peak, and higher-order reflections are observed up to the fifth order. The expected peak positions of the 00L (L = 1 ... 5) peaks are drawn by vertical lines.

The drop-casted films were then thermally annealed above the melting point, and subsequent crystallization of Ph-BTBT-10 was performed at defined cooling rates (Figure 2b). Similarly to the drop-casted films, all the 00L peaks from the annealed samples were clearly visible. However, an outstanding feature is the considerable broadening of the 001, 003, and 005 peaks, while the 002 and 004 peaks do not show broadening with respect to the untreated sample. Peak broadening is more enhanced at larger cooling rates. The largest peak widths of the odd-numbered 00L peaks are observed for the sample prepared using the highest cooling rate of 20 °C/min; in this particular case also, the even-numbered 00L peaks start to broaden.

The next sample series is prepared by thermal-gradient crystallization; the results are depicted in Figure 2c. The 00L Bragg peaks are observed at the expected positions of the known crystal phase. Even in this case, the odd-numbered 00L peaks are broadened considerably in comparison to the even-numbered 00L peaks. No clear dependence of peak broadening is observed as a function of the withdrawal velocity. The last sample series is prepared by bar coating at elevated temperatures from a solution using different solvents. Similarly to the other treatments, the odd-numbered 00L peaks are considerably broadened in comparison to the even-numbered 00L peaks.

An observation of only 00L peaks within a specular diffraction pattern is a frequently observed case^19,31^ and reveals a highly defined preferential out-of-plane orientation of the crystallites. In our case, the crystals grow with the (001) plane parallel to the substrate surface. The appearance of more than a single 001 Bragg peak is referred to higher-order reflections arising from the (001) net plane. The peak width analysis can be performed with high accuracy on the basis of a peak series arising from a series of higher-order reflections. Broadening due to the crystallite size and due to the microstrain can be clearly separated.^32^ However, peak widths that alternate with the order of diffraction cannot be explained by classical diffraction models.^33,34^

**Figure 3:**
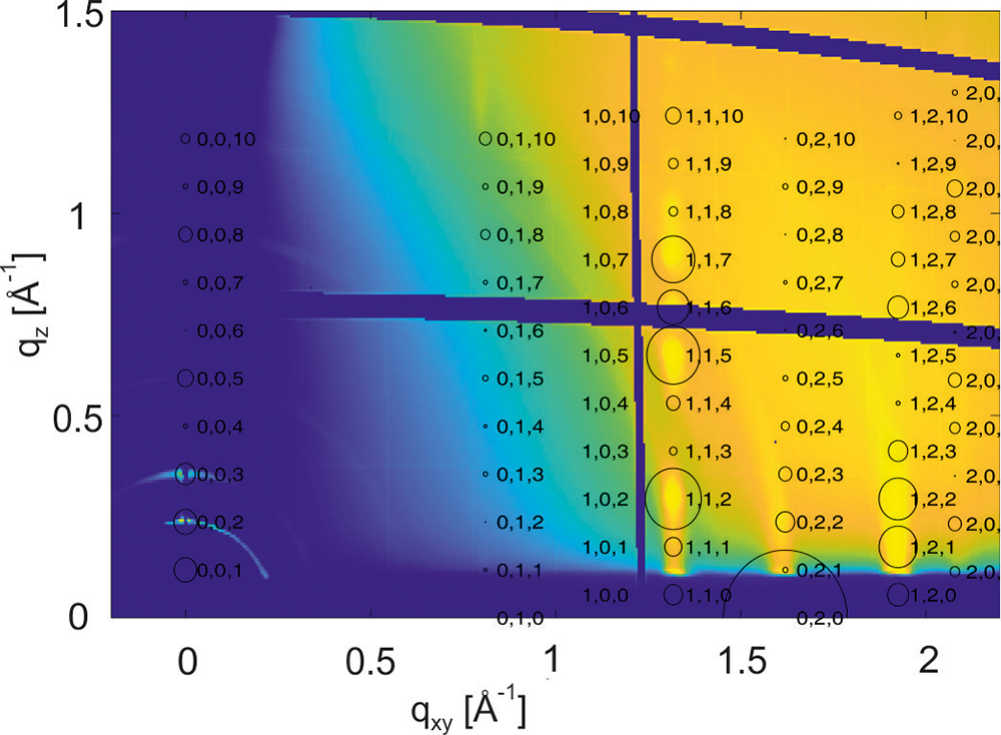
Reciprocal space map of a thermal-gradient crystallized thin film (translation velocity 2.5 μm/sec.) together with the indexation of the diffraction pattern based on the known bulk structure of the molecule Ph-BTBT-10. For indexation, only the strongest Bragg peaks are depicted by circles, where the center and areas of the circles give the peak positions and peak intensities, respectively.

In the next step, more comprehensive structural studies are performed to get more details on the crystallographic properties of Ph-BTBT-10 within thin films. The GIXRD patterns are measured in an extended range so that a characteristic peak pattern of Ph-BTBT-10 is observable. Figure 3 shows a reciprocal space map of a thermally gradient crystallized film. The map shows the characteristic fingerprint of a herringbone packing typically for rodlike conjugated molecules: elongated intensity features are observed along the *q*_z_ direction at defined *q*_xy_ values at 1.31 Å^–1^, 1.60 Å^–1^, and 1.91 Å^-1.^^35,36^ The reciprocal space map is indexed on the basis of the known phase of Ph-BTBT-10, assuming a 001 preferred orientation of the crystallites. The comparison of the calculated diffraction peaks with the experimentally observed peak pattern reveals that the bulk phase of Ph-BTBT-10 is present within the thin film. The excellent agreement can be clearly seen not only for the strongest diffraction peaks like 112, 115, and 117 but also for 121 and 122. The known crystal structure could be confirmed also for the samples prepared by defined cooling rates and for the bar-coated samples (compare Figure S1).

The specular XRD studies (Figure 2) as well as the GIXRD investigations (Figure 3) reveal that the known crystal structure of Ph-BTBT-10 is present within our thin films. Based on the known molecular packing within the bulk structure a possible explanation of the outstanding peak-broadening phenomena can be developed. We assume disorder of the molecules within the crystal structure as the reason for the unusual behavior. The molecular disorder breaks the crystallographic long-range order; therefore, it cannot be treated by classical diffraction theory. As a consequence, statistical simulations have to be performed. The simulation model starts with the crystal structure of Ph-BTBT-10 using double herringbone layers with head-to-head arrangements of the molecules. The molecular disorder is implemented by inverted molecules as deviation from the crystallographic periodicity. The molecular disorder parameter *p* describes the fraction of head-to-head aligned molecules; *p* = 0 represents molecules only with head-to-head arrangement while *p* = 1 represents a molecular packing based on head-to-tail arrangements.

**Figure 4:**
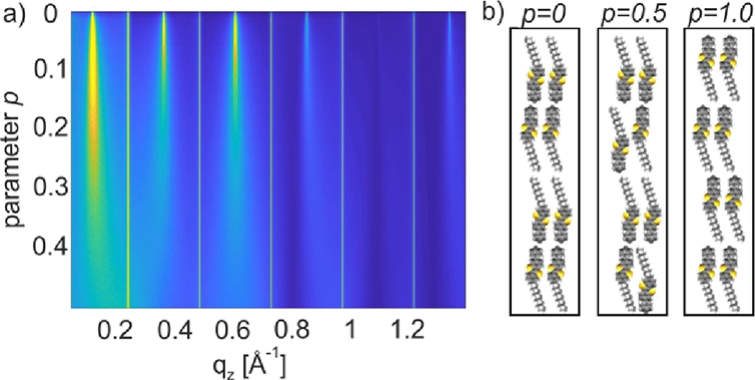
Simulation of a specular diffraction pattern of the (001) plane of Ph-BTBT-10 crystallites with different degrees of random molecular disorder *p* presented in a color contour plot. The intensity scale is color-coded from yellow for high intensity via green to blue for low intensity (a). The molecular disorder parameter *p* represents the deviation from the ideal head-to-head packing present within the known bulk structure (b).

The results of the simulations are depicted in Figure 4. Specular diffraction patterns are plotted as a function of the disorder parameter *p* in a waterfall plot. No peak broadening is observed for *p* = 0, but a slight increase of the disorder parameter immediately causes a peak broadening of the odd-numbered 00L peaks, while the even-numbered 00L peaks remain at their initial peak widths. The peak widths on the odd-numbered 00L peaks increase by increasing the parameter *p* and disappears at about *p* = 0.4. Simulations are performed by random distributions as well as by systematic distributions of the inverted molecules; even random sequences of layers with identical molecular alignment have been considered. We found that the molecular disorder parameter *p* is a reasonable parameter to describe the observed effect on peak broadening.

**Figure 5:**
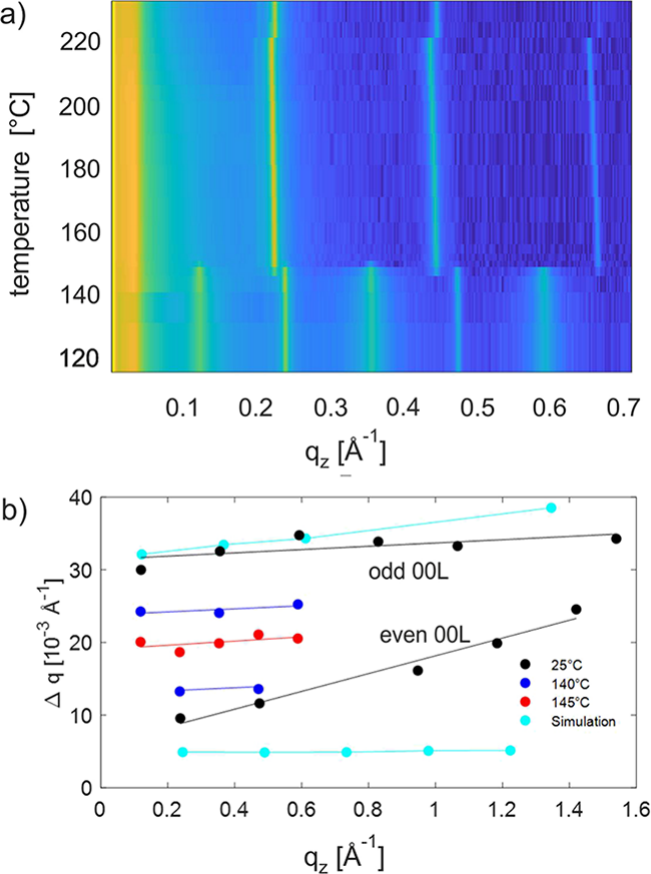
Series of in situ specular XRD of a thermal-gradient crystallized thin film (translation velocity 2 μm/sec.) presented in a color contour plot. The measurements are given in the temperature range from 120 to 180 °C covering the phase transitions from the crystalline state to *smectic E* at 145 °C. The intensity scale is color-coded from yellow for high intensity via green to blue for low intensity (a). Peak width analysis of specular diffraction peaks (Laue indices 00L) of a thermal-gradient crystallized thin film (translation velocity 1.5 μm/sec.) as a function of temperature; additionally, the peak widths from the simulation are given for a molecular disorder parameter *p = 0.15* (b).

In the next step, the influence of heat treatment to the observed peak-broadening phenomena is investigated. Temperature-dependent in situ XRD experiments are performed on a sample prepared by thermal-gradient crystallization. Specular scans were started at 120 °C and performed stepwise in temperatures up to the complete melting of Ph-BTBT-10 at 230 °C. The temperature was increased in steps of 3 °C close to the phase transitions and in steps of 10 °C otherwise. After each increase of the temperature, the system was held at the temperature for 5 min to equilibrate. A section of the measurements is presented using a color contour plot in Figure 5a. The 00L peak series is clearly visible together with their variable peak width (compare Figure 2). At a temperature of 147 °C, the expected phase transition to a *smectic E* phase takes place, clearly visible by the disappearance of the odd-numbered 00L peaks and a clear shift in the peak position of the even-numbered 00L peaks.

The peak widths (Δ*q*) of the in situ studies are evaluated as a function of temperature and plotted in comparison to an ex-situ measurement at room temperature (25 °C). The results are shown in Figure 5b. The plotting of the peak width as a function of the length of the scattering vector q (Williamson–Hall plots) reveals a linear behavior as it is expected from the classical diffraction theory.^33,34^ Considering the peak width at 25 °C, the odd-00L peak series show a different dependence than the even-numbered 00L peak series. Here, the peak widths of the 00L peaks are plotted up to L = 14. In the next step, the peak widths are considered at elevated temperatures. Approaching the phase-transition temperature for the *smectic E* phase, the peak width of the odd-numbered peaks decreases, and the width of the even-numbered peaks increases. At a temperature just below the phase transition at 145 °C, the peak widths of all the 00L peaks are arranged in one line. The variable peak width of the 00L peaks disappears just at the phase transition to the *smectic E* phase. For comparison, we plotted the peak width of our simulation with *p* = 0.15. In both the cases, odd- as well as even-based 00L peaks, we observe a linear behavior. The peak widths are comparable only in the low q-range (for the 001 and 002) because we do not include instrumental broadening in our calculations. Please note that our calculations were performed for a stack of 30 repeating units.

In the final step, the Williamson–Hall plots are prepared for the sample series prepared at defined cooling rates. A clear correlation between the cooling rates and the molecular disorder parameter *p* was found (Figure S2). The cooling rates of 10 and 2 °C/min result in *p* = 0.15 and 0.07, respectively; intermediate rates result in disorder parameters in between.

## Discussion

The thin films were prepared using a variety of methods; in all the cases, a heat treatment procedure was involved. In the first case, the films were obtained by defined cooling from the melt; in the second case, the films were crystallized from the melt within a thermal gradient, and in the third case, the films were obtained by bar coating at elevated temperatures with subsequent cooling to room temperature. After full solidification, the films crystallize in the known bulk structure of Ph-BTBT-10, as shown by specular XRD as well as by GIXRD investigations.

The outstanding experimental observation is that the peak width of the specular diffraction shows a different peak-broadening behavior for the odd-numbered and even-numbered 00L peaks. For an explanation of this effect, a molecular disorder was assumed. Simulations are started with the layered structures of Ph-BTBT-10 with a double herringbone structure with head-to-head-oriented molecules. Gradually introducing defects by inverted molecules, the odd-numbered peaks start to broaden while the even-numbered peaks remain at their initial peak widths. This phenomenon starts already at small deviations from the ideal crystal structure. It is found that the fraction of head-to-head aligned molecules (molecular disorder parameter *p*) is a meaningful parameter which describes the observed effect. Different types of the molecular disorder are investigated. The systematic inversion of all the molecules within one layer or the inversion of random molecules lead to the same result. The assignment of the observed peak broadening to the disorder parameter *p* reveals values of 0.15 for a gradient crystallized sample and values in between 0.15 and 0.07 for samples prepared at different cooling rates.

An interesting effect is that peak broadening disappears as a function of heat treatment. A change in the peak width is observed when the thin film is heated (from room temperature) close to the phase-transition temperature of the *smectic E* phase. Peak broadening disappears fully at the phase-transition temperature, which means that the molecular disorder is strongly reduced. Taking our disorder model into account, the flip-flops of molecules have to appear close to the transition temperature to the *smectic E* phase, which can be explained by a pure translational movement of molecules across different layers.^37,38^

The ability of the molecules to reverse its molecular orientation at elevated temperatures is a known phenomenon. The flip-flop motions of individual molecules are frequently observed in the liquid crystalline state.^39^ Phase transitions (i.e., nematic to isotropic) are analyzed on the basis of flip-flop motions together with the available space of the molecules within the liquid crystalline phases.^40,41^ However, liquid crystalline states with a higher degree of order provides less space for molecular motion, and as a consequence, the molecules are less prone to flip-flop transitions.^42,43^ Also, in the solid state, flip-flop motions become possible but only close to the transition to a liquid state, as observed for the bilayers of phospholipids at the transition from the gel phase to the fluid phase.^44^

## Conclusions

A series of thin films were prepared from the molecule Ph-BTBT-10 using various heat-treatment-based methods and investigated via XRD methods. At room temperature the bulk crystal structure of the material is observed, but a specific anomaly is observed in the peak widths of the 00L Bragg peaks. A model is set up to include a molecular disorder, based on the molecules which are embedded in a reversed manner (flipped molecules) into the bulk crystal structure. The unexplained peak-broadening phenomena can be explained satisfactorily by a disorder parameter, which quantifies the fraction of disordered molecules. It was found that 7 to 15% of the molecules show a reversed molecular alignment because of crystallization from elevated temperatures. The temperature treatment close to the liquid crystalline state allows the molecules to orient into the expected molecular alignment determined by the crystal structure.

## Supporting Information Available

Reciprocal space map of a thin film prepared by a defined cooling rate and that of a thin film prepared by bar coating; peak width analysis of thin films prepared by defined cooling rates compared with the results of peak width simulations (PDF).
