# Supplementary material for: Molecular Disorder in Crystalline Thin Films of an Asymmetric BTBT Derivative
Source: Chem Mater. 2021 Feb 9;33(4):1455–61. doi: 10.1021/acs.chemmater.0c04725 (PMC7905871; doi:10.1021/acs.chemmater.0c04725)
Supplement: Supplementary file 1 — cm0c04725_si_001.pdf [file cm0c04725_si_001.pdf]

Supporting Information

Molecular Disorder in Crystalline Thin Films of an Asymmetric BTBT Derivative

Sebastian Hofer[†], Johanna Unterkofler[†], Martin Kaltenegger[†,‡], Guillaume Schweicher[‡], Christian Ruzié[‡], Adrián Tamayo[$], Tommaso Salzillo[$], Marta Mas-Torrent[$], Alessandro Sanzone[&], Luca Beverina[&], Yves Henry Geerts[‡,§], Roland Resel[†,*]

[†] Institute of Solid State Physics, Graz University of Technology, Austria; [‡] Laboratoire de Chimie des Polymères, Faculté des Sciences, Université Libre de Bruxelles, Belgium; [$] Institut de Ciència de Materials, Universitat Autònoma de Barcelona, Spain; [&] Department of Materials Science, University of Milano-Bicocca, Italy; [§] International Solvay Institutes of Physics and Chemistry Université Libre de Bruxelles, Belgium; [*] corresponding author

The Supporting Information complements the grazing incidence X-ray diffraction investigation presented in the main manuscript. There, one representative GIXD pattern of the sample series prepared by gradient crystallization is shown within Figure 3. Here, representative GIXD pattern of the two other sample series – samples prepared by defined cooling rates and samples prepared by bar coating - are shown.

Additionally, a peak width analysis is performed for the sample series prepared at different cooling rates. The defined variation of the sample preparation conditions allows an assignment of the disorder parameter *p* to the cooling rate of the thin films during the solidification process.

Grazing Incidence X-ray Diffraction

Reciprocal space maps of two different types of thin films are presented just to confirm that in all three types of thin films the known crystal structure of the molecule Ph-BTBT-10 is present. Reciprocal space maps are given for a sample prepared by defined cooling rates and of a sample prepared by bar coating. The results are shown in Figure S1. A stationary measurement was performed in case of the sample prepared with a cooling rate of 5°C / min. The bar coated sample was rotated for 180° with integrating the scattered intensity during the sample rotation. This approach is required, since the bare coating technique induce azimuthal alignment of the Ph-BTBT-10 crystallites.

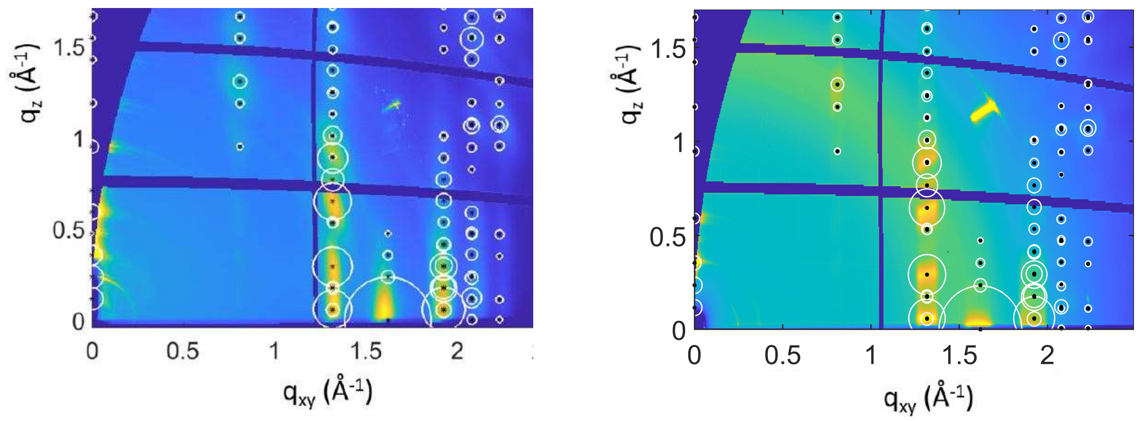

Figure S1: Reciprocal space map of a thin film prepared with a defined cooling rate of 5°C / min (left) and of a bar coated sample prepared from cholorobenzene solution at a temperature of 105°C (right). The circles give calculated peak position / peak intensities from the known bulk structure of Ph-BTBT-10.

Peak width analysis - sample series at defined cooling rates

The peak width of the sample series prepared by different cooling rates are compared with each other. This comparison should reveal the impact of the cooling rate to the molecular disorder parameter *p*. The parameter *p* is found in between 0.07 And 0.15 for the samples prepared at rates of 2°C/ min and 10°C / min, respectively.

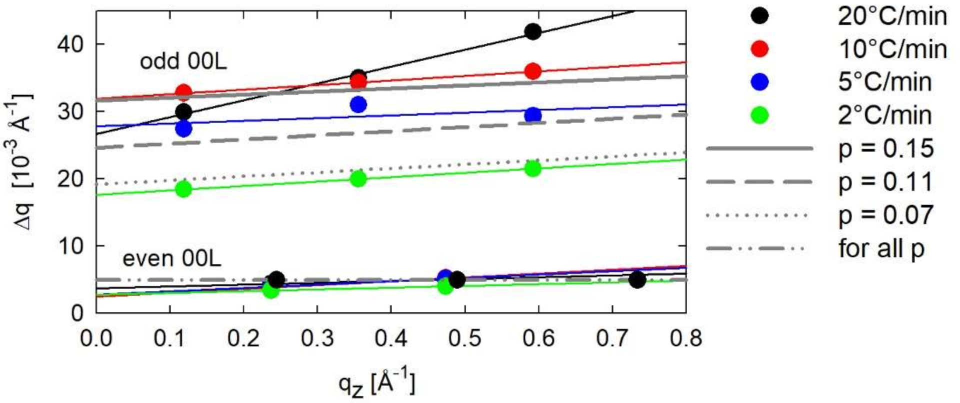

Figure S2: Peak width analysis of the sample series prepared at different cooling rates. Grey lines indicate calculated peak widths at different molecular order parameters *p*.
